# Multifunctional Roles of Extrafloral Nectaries in Shaping Plant–Insect Interactions

**DOI:** 10.3390/plants15040595

**Published:** 2026-02-13

**Authors:** Eduardo Soares Calixto, Renan Fernandes Moura, Denise Lange, Estevao Alves Silva, Helena Maura Torezan-Silingardi, Kleber Del-Claro

**Affiliations:** 1 Entomology and Nematology Department, West Florida Research and Education Center, University of Florida, Jay, FL 32565, USA; 2 Biology Department, University of Cincinnati Blue Ash, Blue Ash, OH 45236, USA; renanfmoura@gmail.com; 3 Postgraduate Program in Natural Resources and Sustainability, Universidade Tecnológica Federal do Paraná, Santa Helena 85892-001, PR, Brazil; deniselange@utfpr.edu.br; 4 Federal Institute of Education, Science and Technology, IF Goiano, Campus Urutaí, Goiás 75790-000, GO, Brazil; estevao.alves@ifgoiano.edu.br; 5 Biology Institute, Federal University of Uberlândia, Uberlândia 38400-902, MG, Brazil; hmtsilingardi@gmail.com; 6 Biology Department, University of São Paulo, Ribeirão Preto 05508-090, SP, Brazil

**Keywords:** distraction hypothesis, ecological factors, evolutionary ecology, mutualism, plant-ant interactions, pollination, protection hypothesis, herbivory, continuum hypothesis

## Abstract

Understanding the net outcomes of ecological interactions by examining the costs and benefits of organism associations is central to ecology. The mutualistic relationship between ants and plants mediated by extrafloral nectaries (EFNs) has long been viewed as protective, with ants defending plants from herbivores in exchange for nectar. However, alternative hypotheses, like the ant-distraction and flower-distraction, highlight the multifunctionality of EFNs. The flower-distraction hypothesis proposes that EFNs evolved to divert ants from flowers, reducing ant impact on pollination. Recent studies reveal that EFN interactions with ants are highly context-dependent, shaped by factors such as EFN location and ant behavior. Although EFNs often occur on vegetative tissues, they are sometimes located near flowers, raising the possibility that they serve both protective and distracting roles. This duality challenges the notion that EFNs can be categorized exclusively by location or function. Instead, their ecological roles likely shift in space and time, depending on plant growth form, pollination system, and interacting species. We propose moving beyond a dichotomous framework toward a nuanced perspective that embraces a potential continuum of functionalities. Considering multiple ecological and evolutionary factors will enhance understanding of EFN evolution, plant–animal interactions, and ecosystem dynamics.

## 1. The EFN Functionalities

**Figure 1:**
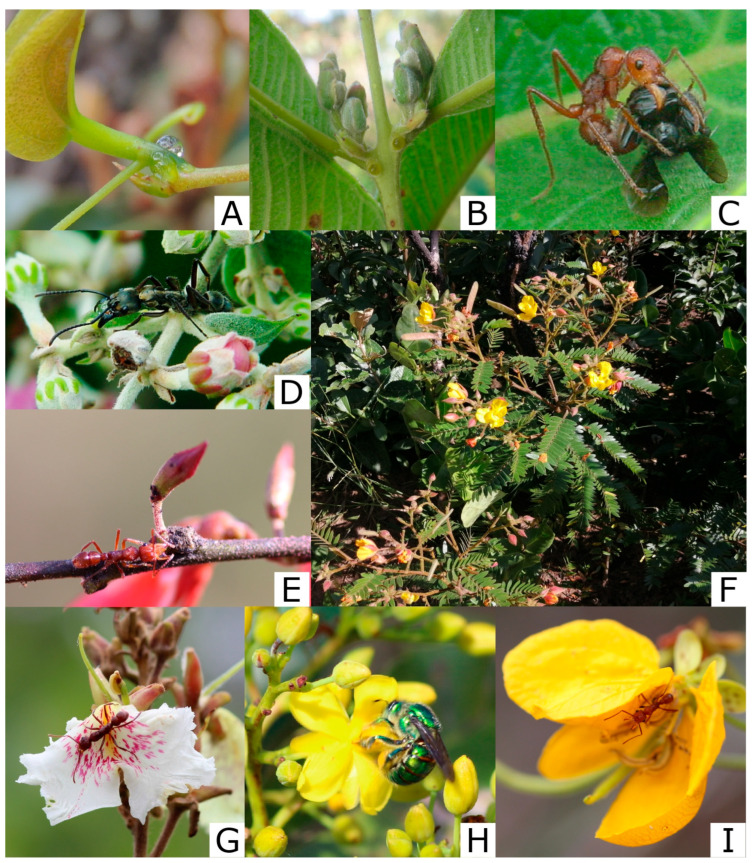
(**A**) Extrafloral nectar produced by the EFNs of *Smilax polyantha* (Smilacaceae). (**B**) EFNs located on the reproductive structures of *Qualea multiflora* (Vochysiaceae). (**C**) *Ectatomma tuberculatum* preying upon *Trigona* sp. on *Banisteriopsis malifolia* (Malpighiaceae). (**D**) *Neoponera villosa* collecting extrafloral nectar and foraging on the reproductive parts of *B. malifolia*. (**E**) *Ectatomma tuberculatum* foraging on EFNs located on the reproductive structures of *Bionia coriacea* (Fabaceae). (**F**) *Chamaecrista desvauxii* (Fabaceae), a shrub from the Brazilian savanna (Cerrado). (**G**) *Ectatomma tuberculatum* foraging on the flowers of *Q. multiflora*. (**H**) Bee collecting pollen from the buzz-pollinated flower of *Ouratea spectabilis* (Ochnaceae). (**I**) *Ectatomma tuberculatum* foraging on a flower of *Senna* sp., a buzz-pollinated plant (Fabaceae).

Mutualism is among the most ubiquitous types of ecological interaction on Earth. It is essential for understanding key aspects of a species, e.g., its natural history and behavior, and it also helps explain broad ecological patterns, including species’ geographic distributions and range limits [1,2]. Indirect defenses (also known as biotic defenses) involving extrafloral nectaries (EFNs) are a classic example of animal–plant mutualisms (Figure 1A–E). In these interactions, plants produce sugary rewards for defensive partners such as ants, wasps, and spiders, which, by consuming the nectar, acquire resources that enhance their colony fitness [3,4,5]. EFNs occur in more than 4900 species across 130 families distributed worldwide [6,7]. Many studies have shown that EFNs significantly affect both plants and their mutualistic partners. For instance, meta-analyses have demonstrated that the reproductive output of plants increases by about 50% and herbivory levels decrease by 62% in the presence of mutualistic ants [8,9]. While most benefits to plants have historically been attributed to the indirect defenses provided by ants or other animals, EFNs can support plants in multiple ways. In this study, we review the main mechanisms and factors underlying the ecological functions of EFNs, and we propose moving beyond the traditional dichotomous and categorical view of EFN functionality toward a more nuanced perspective, where EFN functionality should be treated in a continuum perspective.

Different hypotheses have been proposed to explain the evolution and functionality of EFNs. In the classic protection hypothesis (also known as the ant-guard hypothesis), ants and plants engage in a mutualist interaction where plants offer sugary rewards to ants (Figure 1A) and plants are protected by ants against herbivores. This hypothesis has been extensively investigated [10,11,12,13] and contributed to the common view that EFNs have a well-defined function of indirect defense. Although ants are the main attracted organisms and the primary group of plant bodyguards, other arthropods (e.g., spiders, wasps, and mites) may provide a supporting defensive role [14,15]. Researchers, however, came to perceive the protection hypothesis as insufficient, as observations and experiments eventually failed to support it [8,9]. Some negative results occur, as certain visiting ant species are physically or behaviorally unable to prey on or drive away herbivores [16]. Other factors include the presence of parasitic ants, low ant visitation, the nutritional conditions of ants, reduced nectar secretion during certain phenological stages, and the presence of herbivores that can either avoid ants or retaliate [17,18,19]. Therefore, researchers have proposed alternative explanations for the functionality of EFNs (Table 1), although some hypotheses are stricter than others. The ant-distraction hypothesis, for instance, states that EFNs can satiate and distract ants from protecting honeydew-producing insects, thus minimizing herbivore damage [20]. However, this hypothesis cannot explain the ecological role of EFNs in most systems due to its dependency on a specific herbivore type [11]. An alternative hypothesis that requires fewer specific factors is the flower-distraction hypothesis. This hypothesis has received consistent support [21,22,23,24], and states that EFNs can lure non-pollinator species away from flowers, mitigating possible conflicts with pollinators (Figure 1C) and potential damage to reproductive structures [22,25]. Based on the extensive information available on the protection hypothesis and the growing evidence supporting the flower-distraction hypothesis, here we will focus on discussing and expanding ideas related to how EFN functionality relates to the protection and distraction functionalities, and how both abiotic and biotic factors may influence it.

It is hypothesized that the function of EFNs depends on their location on the plant. In a recent meta-analysis, researchers showed that the effectiveness of ant–plant protection mutualisms is contingent on both EFN location on plants and ant aggressiveness [26]. Although EFNs are usually found on vegetative parts (henceforth vegetative EFNs), for instance, leaves (Figure 1A), many plant species have these glands on or very close to reproductive parts (henceforth reproductive EFNs), including bracts, sepals, and peduncles/pedicels of inflorescences [27,28,29,30,31,32] (Figure 1B,D,E). Indeed, reproductive structures are associated with plant fitness, and reproductive EFNs can provide indirect protection against herbivores through ant attraction [31,33,34,35]. For instance, Del-Claro et al. [33] observed a significant reduction in damage levels of buds and flowers of *Qualea multiflora* when ants were present. However, ant bodyguards are often not welcomed to reproductive parts since they can disrupt pollination processes [24,30,36,37,38,39,40,41,42]. For instance, some ants can reduce the number and viability of pollen due to metapleural secretion, reduce the quantity and quality of floral nectar available to pollinators, and dissuade or even prey upon pollinators (Figure 1C), potentially causing negative effects on plant fitness [36,43,44]. Thus, plants may avoid the potential negative impacts of ants on plant reproduction via reproductive EFNs (Figure 1B,D,E) as a distraction mechanism (flower-distraction hypothesis).

Nectar concentration also plays a critical role in mediating ant behavior in many plant species, as the nectar composition can influence ant foraging behavior more than the shape or location of EFNs on the plant [45,46,47,48]. In a system involving carpenter ants, particularly *Camponotus renggeri* and *Qualea multiflora* (Vochysiaceae) plants, we have shown that EFNs on reproductive parts are richer in sugar than those on vegetative parts [35]. EFNs that produce highly concentrated nectars tend to attract more ants, reducing the time they spend patrolling the plant (ESC unpublished data), thereby supporting the flower-distraction hypothesis. In another study testing the predatory effectiveness of *Camponotus crassus* foraging on the vegetative EFNs of five EFN-bearing plant species in the Brazilian savannah, we found high predation rates on surrogate herbivores (termites) in plant species that produced greater volumes and higher sugar concentrations of nectar [48]. This idea aligns with the concept that EFNs, as an indirect defense mechanism, should be attractive enough to maintain predatory ants on plants, but not so abundant or nutritionally complete as to keep ants satiated and unwilling to move around the plant [19].

Most studies have tested and supported the protection and flower-distraction hypotheses independently [8,9,22,24,26], which has improved our understanding of ant–plant interactions, but are still largely divided between these two perspectives. However, we argue that EFNs can be involved with multiple functions; in other words, the two main hypotheses are not necessarily mutually exclusive, and perhaps are more complementary than previously thought [49]. Although the idea that EFNs may be multifunctional is generally acknowledged, this has typically been addressed by comparing different EFN systems (e.g., the protection effectiveness of vegetative EFNs versus reproductive EFNs [35], or the protection effectiveness of vegetative EFNs in one species versus vegetative EFNs in another species [48,50]), rather than examining multiple functions within the same EFN system (e.g., protection versus flower-distraction in a reproductive EFN [24,49,51]). As a result, EFN multifunctionality has rarely been explicitly incorporated into hypothesis testing or conceptual frameworks, which has kept the literature largely dichotomous. In certain ecological contexts, some limited evidence suggests that EFNs can assume both protection and distraction functions and provide additive benefits to the plant [24,49,51]. In fact, having the same structure acting as an indirect defense mechanism and as a way to prevent ants from foraging in flowers could prove, in some ecological contexts, the most parsimonious and evolutionarily stable strategy for plants. Thus, our aim is to show that the evolutionary function of EFNs should not be evaluated “dichotomously” (e.g., either protection or distraction), as is commonly done [8,26], but in a continuum, with possible asymmetric, additive, or even synergic effects, considering different ecological factors operating in each evaluated system [49,51,52]. Below, we discuss some factors that potentially shape the functions and effectiveness of EFNs, and propose a new hypothesis for the functionality of EFNs, which is the “continuum hypothesis” [52,53].

**Table 1:** The main hypotheses concerning the functionality of extrafloral nectaries (EFNs). The continuum hypothesis proposed here states that EFNs can have multiple functions depending on different factors, e.g., the identity of the organism involved in the interaction. F and EF nectar mean floral and extrafloral nectar, respectively.

EFN Function	Description	Predominant Type of EFN	Factors to Be Considered
Protection hypothesis [54]	Defense against herbivores	Vegetative	Ant species and behavior, herbivore species, herbivore population/community, herbivore damage, plant phenology, EF nectar production, plant size, growth form, neighboring plants, plant fitness
Flower-distracting hypothesis [21,22]	Distracting ants from flowers	Reproductive	Ant species and behavior, pollinator species and specialization, ant damage, plant phenology, floral morphology, reproductive system, F and EF nectar production, plant size, growth form, neighboring plants, plant fitness
Ant-distracting hypothesis [20]	Distracting ants from honeydew-producing insects	Vegetative/reproductive	Ant species and behavior, herbivore species, herbivore damage, herbivore and plant phenology, EF nectar production, plant size, growth form, neighboring plants, plant fitness
Herbivore-distracting hypothesis [55]	Distracting chewing herbivores from vegetal structures	Vegetative	Ant species and behavior, herbivore species, herbivore population/community, herbivore damage, plant phenology, EF nectar production, EFN number, plant size, growth form, neighboring plants, plant fitness
Exploitation hypothesis [56]	EF nectar is simply a byproduct of plant metabolism	Vegetative/reproductive	EF nectar production, plant homeostasis, photosynthetic rates, growth form, plant fitness
Continuum hypothesis [52,53]	EFNs may have multiple functions with possible asymmetric, additive, or synergic effects on the plant	Vegetative/reproductive	All factors described above

## 2. Understanding EFN Functionality Through Organism’s Traits and Interactions

A plethora of factors can be related to the functionality and effectiveness of EFNs, including the spatial location of the EFNs, the growth form and reproductive system of the plants, and the associated species (e.g., pollinators, herbivores, and mutualists) (Table 2). Due to the complexity of EFN-mediated systems, a useful approach is to first investigate and decipher their parts, which include the fundamental natural history aspects of the involved ant, pollinator, herbivore and plant species. This initial investigation is particularly important given the contingent nature of EFN-mediated systems [57,58] and the tremendous variation among plant and animal traits.

### 2.1. Plant Traits

Species that share specific growth forms (e.g., shrubs, trees, lianas) [59] illustrate our idea of a functional gradient well. Due to their smaller size, shrubs, vines, and other small plants can be entirely patrolled by ants (Figure 1F), including their reproductive parts [23,60]. Therefore, ant–pollinator interactions may be more likely to occur in small plants, suggesting that EFNs might represent an important distraction mechanism in these plants, even though EFNs are located on vegetative parts. Evaluating the impact of invasive ants on the floral visitors and fruit set of *Calystegia macrostegia* (Convolvulaceae), a plant not bearing EFNs, Hanna et al. [60] observed that the presence of ants can significantly disrupt the pollination services, negatively affecting plant reproduction. Considering plants bearing EFNs, studies have shown that the presence of EFNs on smaller plants like shrubs and vines can lure away ants from flowers, or even have a synergistic effect of protection and distraction [49]. Studying the shrub *Turnera velutina*, Villamil et al. [23] showed that the vegetative EFNs in this plant species can significantly reduce the occupancy of ants on flowers, supporting the flower-distraction hypothesis, even though the EFNs are located on vegetative parts. Chamberlain and Holland [25] showed an increase in the aggregate density of ants in the EFN-bearing senita cacti (*Pachycereus schottii* Engelmann), but a decrease in the number of ants in the floral vicinity, also supporting the flower-distraction hypothesis. Also working with the senita cacti, Holland et al. [49] observed that EFNs may not only distract ants from disrupting plant–pollinator interactions, but they may also increase pollination and reduce wasp parasitism, enhancing plant–pollinator interactions. Results from Holland et al. not only support the distraction hypothesis but also the protection hypothesis, providing evidence that the functions of EFNs are not mutually exclusive, but rather synergistic in some plant systems (Table 1).

Large plants, including trees, tend to have high numbers of EFNs [61]. These EFNs can be located on vegetative, reproductive (Figure 1B), or both parts of the plant, and they may be active during different stages of plant ontogeny [31,62]. For instance, *Qualea multiflora* (Vochysiaceae) is a Brazilian savannah tree with EFNs on both its vegetative and reproductive parts (Table 2). When the EFNs on the vegetative parts are active, the plant is not producing flowers, which supports the protection hypothesis [35,50]. When plants begin to bloom, vegetative EFNs stop producing nectar, and reproductive EFNs become active. Del-Claro et al. [33] observed a decrease in herbivory on reproductive parts and an increase in fruit set in this plant species, supporting the protection hypothesis. Calixto et al. [52] showed that active EFNs on the reproductive parts of *Q. multiflora* reduce the number of ants foraging on flowers (Figure 1G), supporting the flower-distraction hypothesis. These findings corroborate our proposed continuum hypothesis, where EFNs can perform multiple functions (Table 1), that is, providing protection and distracting ants. The implications of plant size on EFNs evolution cannot be taken in isolation, but rather, they should be considered in combination with other components from any system (see below).

In contrast to *Q. multiflora*, several species of tropical shrubs, particularly those in Malpighiaceae (Table 2), possess EFNs on their leaves (vegetative EFNs) and on the bracts (reproductive EFNs) along inflorescences [63] (Figure 1D). As a result, patrolling ants frequently visit flowers [64]. However, this ant activity did not affect bee visitation to the flowers of *Banisteriopsis malifolia* [64], and in *Heteropterys pteropetala,* no significant reduction in fruit set was observed due to the presence of ants [40]. In fact, ant presence on the flowers of *B. malifolia* reduced visits from non-pollinating bees, thereby decreasing interspecific competition and ensuring that plants received more visits from effective pollinators [64].

The nature of EFNs in Malpighiaceae supports the continuum hypothesis (Table 1). EFNs located along inflorescences ensure that ants will forage across virtually all plant structures, thereby deterring herbivores from multiple niches and reinforcing their role as bodyguards (i.e., protective hypothesis). However, ants are particularly effective against folivores [65]. When ants fail to exclude herbivores from flowers, EFN-feeding wasps compensate by providing additional protection [66,67,68]. Thus, both predators act synergistically as defensive partners. EFNs, per se, do not prevent ants from visiting flowers in this system, as predicted by the flower-distracting hypothesis [16]. However, as Malpighiaceae flowers do not produce nectar, the presence of ants in these structures likely results from their high foraging activity and exploratory behavior, particularly among *Camponotus*, the main partner of Malpighiaceae [51,63]. Although ant visits to flowers and their aggression toward pollinators [41,53] may constitute collateral costs, the overall benefits of ant protection appear to outweigh these negative effects, favoring the persistence of the interaction. Finally, the EFNs of *B. malifolia* distract ants from tending myrmecophilous hemipterans, giving further support to the ant-distracting hypothesis [51]. Malpighiaceae, along with other shrubs that possess reproductive EFNs [45,69,70], represent promising models for further investigation of the continuum hypothesis. Comparative studies with tree species in which EFNs are restricted to leaves and not associated with reproductive structures (e.g., *Inga* spp.) [71] could clarify how some plant traits influence the functional role of EFNs [72].

### 2.2. Ant Species

The ant species involved in the mutualistic plant-ant system is a key factor in understanding the functionality of EFNs. Visiting ant species strongly vary in space and time, and within and between plant individuals, given multiple environmental factors, including EF nectar production [50,73]. Many studies have demonstrated that the spatial location of food resources can affect the recruitment and behavior of visiting ants, and consequently, their protection of plants [53,74]. Hence, it is crucial to comprehend the sensorial and mobile abilities of ants, which vary tremendously. While some species travel less than a dozen meters a day, others can travel hundreds of meters, even in dense and difficult terrains [75,76,77]. Naturally, ants foraging for EF nectar vary in size, aggressiveness, and protective effectiveness [37,46,48], which directly affects the EFN functionality. Elusive and docile ant species have little or no effect on plant defense but can still cause negative impacts on pollination processes [37,39,52,53]. It is possible that EFNs, in this case, are more related to distraction than protection [53]. In systems where protecting ants are extremely aggressive, EFNs may also be involved with distraction, since aggressive behaviors could result in negative effects on pollinators [37,53,60] (Figure 1C). Highly aggressive ants also tend to monopolize resources and attack other ant species, decreasing visiting ant diversity [78,79]. Hence, the functionality of the EFNs might be related to the balance between the positive effects of protection against herbivores and the negative effects on the pollination process [53,80]. The interference of ants on pollination is complex and nuanced. Although ants may reduce the visitation time of pollinators per plant, they may eventually force pollinators to visit an increased number of individuals [81], therefore resulting in lower levels of self-pollination [30,42,52,53]. These ideas have yet to be tested, including the possibility that plants can evolve other ways to cope with the negative effects of ant visitation, including floral repellents and temporal segregation between pollinator visitation and ant activity [53,82,83].

### 2.3. Pollination Systems

Multiple aspects of flowers and pollination systems can provide insights into the ecological role of EFNs. For example, about 80% of all angiosperms rely on animals to perform pollen transfer [84], while the remaining species rely on other mechanisms (e.g., self-pollination, wind pollination). When plants show little dependence on pollinators to reproduce, less selective pressure is expected to direct EFNs towards distraction mechanisms. Thus, EFNs of plants showing predominant asexual reproduction mechanisms (e.g., clonal propagation, apomixis) could be mostly related to protection, given that they do not often require pollination services [53]. Furthermore, mitigating herbivory damage via indirect defenses may be particularly important for clonal plants since they generally have reduced genetic variability and are sensitive to environmental stress [85,86,87]. The available evidence, however, shows that clonal plants invest less in defenses [88], and that might include indirect defenses, as these plants commonly rely on fast reproduction and development times [89,90]. Hence, we hypothesize that EFNs are uncommon in this group. This matter should be further investigated and tested, but this initial assessment indicates that reproductive systems may play an important role in the evolution of EFNs. An interesting natural experiment would be a comparison between two sympatric species with EFNs manifesting distinct reproduction mechanisms (sexual and asexual).

Flower rewards may affect EFN functionality. For instance, some plants can produce floral nectars that are toxic to nectar robbers, including ants [91]. Given that the toxic nectar can be a defensive mechanism by itself, distraction mechanisms concerning EFNs would be redundant and perhaps less important in this context. Additionally, large nectar-consuming pollinators (e.g., bats, hummingbirds) are less affected by the ant presence [32,92], even when ants act as nectar robbers. Caballero et al. [93] found that ants reduced the nectar availability of a hummingbird-pollinated plant, *Tristerix aphyllus* (Loranthaceae), by only 8%, with no noticeable negative effects on hummingbird visitation. In addition, Cardoso et al. [32] found that large and very aggressive ants reduced the fruit set of the hummingbird-pollinated plant *Palicourea rigida* (Rubiaceae), but medium-sized and less aggressive ants did not. These results show that EFN functionality is related to a plethora of factors, including flower rewards and visitors.

Regarding floral morphology, consider poricidal anthers (Figure 1H,I), which are tube-shaped structures with small pores used to release the pollen. These anthers usually require a special pollination process called buzz-pollination [94], where bees need to employ complex and unique vibrational patterns to release the pollen [95,96,97]. This process is time and energy-consuming [98], and a study suggested that buzz-pollinated plants are weak competitors, as bees may prefer visiting flowers with easily accessible pollen [99]. Poricidal anthers are structurally tough due to the presence of calcium oxalate crystals and a thick endothecium [100,101], which may reduce pollen theft by herbivores [102,103]. In a context where flower structures require less defense and where pollinators are particularly sensitive to the presence of aggressive ants, EFNs could be hypothesized to be more related to ant distraction than protection.

Pollinator diversity is largely attributed to diverse floral morphologies and floral rewards (e.g., nectar) [104]. The anatomical and behavioral traits of pollinators must fit the traits of the flowers they visit, as this match influences their ability to effectively remove and deposit pollen [105]. Social bees (e.g., *Apis mellifera*) are usually considered generalists, whereas solitary bees (e.g., *Bombus* spp. and *Xylocopa* spp.) are often associated with specialized floral structures [96,106]. Flower rewards from highly specialized plants can be difficult to access, requiring specialized pollinators, which are selected, in part, by species offering high-quality floral rewards [107]. These highly specialized flowers often impose chemical or physical barriers to exclude not only inefficient pollinators but also non-pollinator visitors, for instance, ants [108,109]. Pollination in such systems is complex, requiring precise conditions to ensure pollen transfer. Morphologically complex flowers restrict access to nectar and pollen and prevent interference by unwanted visitors (e.g., ants), which can disrupt pollination and negatively impact plant fitness [110,111]. In this scenario, only certain visitors with appropriate morphological and behavioral traits related, for instance, to body size [52], abundance, or pollination behavior (e.g., buzz versus non-buzz pollination) can have access to plant resources [96,112,113]. Buzz-pollinated flowers exemplify a complex pollination system (Figure 1H,I). These flowers are usually adapted to the size of their pollinators, and pollen release depends on a specific vibration frequency, which requires the pollinator to spend more time on the flower to complete the vibration process [97]. In these cases, even slight disturbances can impair pollination efficiency and reduce plant reproductive success.

Another example of a specialized pollination system involves the EFN-bearing tree *Qualea multiflora* (Vochysiaceae). The flowers of this plant have only one stamen and one pistil, both elevated, restricting pollination to individuals capable of contacting both structures (Figure 1G). Nectar is available in a spur, further limiting access to both nectar collection and simultaneous contact with pollen and stigma. Once open, flowers remain viable for only 24 h, sometimes up to 48 h [52,114,115]. Therefore, for successful pollination, pollinators must fit a specific size to touch these parts, typically bumble bees and carpenter bees [52,114,115]. Since these groups of bees are less abundant and visit plants infrequently [110], this reduces the potential for successful pollination. All these factors make this a complex pollination system, where any ant interference can disrupt the process. Due to the presence of EFNs on reproductive parts, this suggests that EFNs in complex systems like that may serve more to distract ants than to protect the plant from herbivory. Although traits of specialized flowers may affect EFN functionality by mitigating direct pollination interference by ants, it is important to observe that ants are one of the main insect groups involved with nectar robbery [116]. Countless factors related to the pollination system could be included here, such as anthesis, the number of produced flowers, flower morphology, the location and availability of floral nectar, and the type of pollinators, to name a few. When all these factors are combined, we expect that the more complex the pollination system, the more likely EFNs are to align with the distraction hypothesis.

### 2.4. Herbivores

Herbivory patterns (e.g., type of herbivore, sites of damage within the plant, levels of damage) also can offer great support for understanding the role of EFNs. For example, one would expect that plants that are attacked more frequently and/or with high levels of damage would have EFNs more related to protection than distraction. As predicted by the optimal defense hypothesis [35,117], many plant species respond to herbivore attacks by increasing the volume and concentration of EF nectar to attract more ants [34,118]. On the other hand, in cases where plants are not frequently attacked and/or do not suffer as much damage, EFNs could be hypothesized to be less related to protection. However, understanding herbivory patterns also depends on the herbivore–ant relationship. While visiting ants can offer effective protection against a particular type of herbivore (e.g., cricket, beetles, caterpillars), they might be ineffective against others due to key morphological and behavioral traits [16,46]. The presence of herbivores does not always result in a deficit to the plant. Therefore, when herbivore damage is negligible, a constitutive investment in EFNs would represent an unnecessary energetic cost for the plant. In such a context, EFNs could be induced or associated with other functions than protection. A less explored form of distraction suggests that EFNs can distract herbivores from damaging flowers and leaves. Kirmse & Chaboo [55] found that nearly 20% of the sampled beetle species from a Venezuelan Amazonian rainforest were observed feeding on EFNs. They suggest that plants offering EF nectar to herbivore beetles would reduce their necessity to consume plant structures. That may be advantageous to the plant since producing EF nectar is not costly [119] and requires just a fraction of the energy necessary to produce structures like leaves [120]. Regardless, if plant fitness is unmeasured, merely observing whether herbivory levels change with the ant presence is not enough to make accurate predictions. For example, high herbivory can have little effect on fruit production in a certain plant, but the plant may still exhibit low production due to an ant–pollinator interference. In that case, the environmental pressure on EFNs would likely lead them to distraction mechanisms rather than protection.

## 3. Future Research Directions

### 3.1. Phylogenetic and Comparative Approaches

A valuable direction for future research is to investigate whether EFN functionality along the continuum, ranging from primarily protective to primarily distracting roles, shows a phylogenetic signal. Comparative phylogenetic analyses across the >130 plant families (or across plant genera) that bear EFNs could clarify whether EFN functionality has evolved repeatedly or whether certain clades favor particular functional combinations [6]. For example, phylogenetic comparative analysis could be used to test whether EFNs with protection or distraction functions cluster in specific lineages or whether multifunctionality evolves opportunistically in response to ecological pressures. Understanding these patterns would help predict where multifunctional EFNs are most likely to be found and what ecological pressures shape transitions along the EFN continuum.

### 3.2. The Plant Perspective

Another important direction is to examine EFNs explicitly from the plant’s perspective, with a focus on energetic costs, tissue allocation, and trade-offs with other defenses [53]. Plants likely experience different fitness outcomes depending on where an EFN falls on the protection–distraction continuum in a given environment. Experimental manipulations, such as EFN suppression, inducibility, or resource-limitation treatments, could help quantify the costs of producing nectar versus the benefits accrued from ant activity or pollinator services [31,118,121]. By linking EFN function to plant performance, studies could better evaluate when multifunctionality is favored and when plants benefit from EFNs that specialize in a single role.

### 3.3. Integrating Ant–Hemipteran Interactions into EFN Theory

Insights from ant–hemipteran mutualisms offer a promising parallel that can strengthen our understanding of EFN evolution and function [122,123]. Hemipterans can be considered “animal equivalents of EFNs,” as they provide sugar rewards to ants and receive protection in return. As highlighted in a recent study [122], hemipteran traits can shape their interactions with ants and the consequent effects on host plants. A similar trait-based framework could be applied to EFNs. For instance, variations in nectar composition, timing of secretion, volume produced, and EFN location may predict the strength or direction of ant responses. Comparative studies examining plant systems that simultaneously host EFNs and hemipterans could reveal whether EFNs function to attract protective ants or to distract them from tending honeydew-producing insects [123]. Incorporating this perspective would provide a more holistic understanding of ant-mediated plant defense and clarify how EFNs fit into a broader set of plant strategies.

### 3.4. Molecular Analysis and Chemical Ecology

Another important step would be identifying the genetic mechanisms underlying EFN development, activation, and spatial distribution on plants [13]. Studies examining gene expression across tissues and ontogenetic stages could reveal how EFN production is regulated and whether certain genes are associated with multifunctionality. For example, transcriptomic or qPCR analyses could evaluate whether EFNs that have both protection and distraction functions show distinct expression profiles compared to EFNs that have a single role. Studies could also determine whether EFN functions shift over the life cycle. For instance, whether young leaves favor protective EFNs while reproductive stages favor distraction functions, as described by Calixto et al. [53]. Insights into the genetic background related to regulating sugar production, secretion pathways, or inducible activation would help clarify how plants modulate EFN function in response to ant activity, herbivore pressure, or pollinator service.

In addition, the chemistry of EF nectar offers another rich area for future study. EFN rewards consist not only of sugars but may also include amino acids, lipids, secondary metabolites, and volatile organic compounds [124,125,126]. These components can influence ant species composition, visitation rates, aggressiveness, and even ant hierarchies [45,47,48]. Improved methods for detecting and quantifying these compounds (e.g., metabolomics, gas chromatography associated with mass spectrometry for nectar compounds) could reveal chemical particularities associated with different EFN functions. For example, increased amounts of sugar can increase ant aggressiveness and protective effectiveness [47,48], but very high sugar concentrations can decrease ant patrolling and protection effectiveness (ESC unpublished). Understanding the EF nectar composition and the biosynthetic pathways behind these compounds will further clarify the function of EFNs in the protection–distraction continuum.

## 4. Concluding Remarks

**Figure 2:**
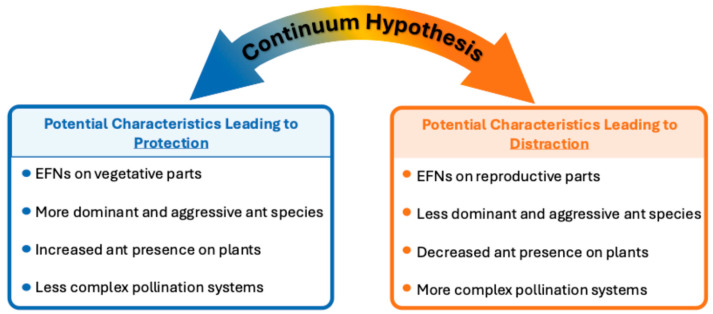
Conceptual diagram of the continuum hypothesis. Traits on the left illustrate conditions that typically favor protective functions of extrafloral nectaries, while traits on the right reflect conditions that can favor distraction from reproductive structures. The gradient arrow highlights that EFN functions vary along a continuum rather than fitting a strict protection-versus-distraction dichotomy.

Evaluating the evolutionary and ecological role of EFNs is a complex task. These glands are diverse in morphology and distribution on plants, produce nectars of contrasting quality, and interact with a plethora of organisms [127]. The functionality of these structures can be unique in some systems or multipurpose in others, as proposed here. How to classify and understand the ecological roles of EFNs in plants, then? We believe that the best approach would be to investigate the costs and benefits of multiple factors related to EFN-bearing plants, including ant–herbivore interactions and pollination processes from distinct plant systems [53], as discussed in this review (Figure 2). Indeed, many other undiscussed factors can be harnessed to appreciate the functionality of EFNs, including vegetation systems (e.g., temperate versus tropical systems, regions with high productivity versus low productivity, forests versus savannas) and their historical and biogeographical aspects. We believe that it is necessary to move towards a more quantitative approach and further investigate the plasticity of EFNs, as they may exhibit multiple and complementary functions that vary in space and time and even within species. A complete understanding of the role of EFNs could help explain their convergent evolution across 130 plant families, their widespread ecological success as mutualistic structures, and their positive impact on biodiversity.

## Figures and Tables

**Table 2:** Examples of botanical families with different plant forms, EFN locations, micromorphological traits, and/or secretion modes. Importantly, each species, even within the same genus, may exhibit distinct characteristics; therefore, conclusions regarding EFN functionality should be made on a species-by-species basis.

Family	Plant Form	Representative Species	Location of EFNs	Micromorphology/Ultrastructure	Nectar Secretion Mode
Bignoniaceae	Woody vine	*Arrabidaea brachypoda*	Leaf surface near midvein (adaxial/abaxial)	Scale-like EFNs; flattened or slightly raised disks; secretory palisade epidermis; subglandular layer of thick-walled isodiametric cells; surrounded by peltate secretory trichomes	Secretion released directly through epidermal surface; accessible to ants
Fabaceae	Shrubs/herbs	*Bionia coriacea*; *Chamaecrista* spp.; *Senna* spp.	Leaflets, rachis, stipules; sometimes reproductive structures (bracts/inflorescences)	Raised glandular pads or domes; secretory epidermal cells; underlying parenchyma; EFNs often associated with high sugar content	Epithelial (granulocrine) secretion; often diurnally variable
Malpighiaceae	Shrubs/lianas	*Banisteriopsis stellaris*	Abaxial leaf surface and petiole	Discoid elevated nectaries; palisade secretory epidermis; subepidermal parenchyma vascularized by phloem + xylem; cuticle often distended or wrinkled, indicating nectar accumulation	Secretion via cuticular pores into subcuticular chambers
Rubiaceae	Shrub/small tree	*Palicourea rigida*	Pericarpial EFNs on developing fruits (post-floral nectaries)	Cup-shaped or disk-shaped secretory tissues surrounding gynoecium/fruit base; thickened epidermis; nectary development synchronized with fruit phenology	Epithelial secretion correlated with reproductive stage
Vochysiaceae	Tree	*Qualea grandiflora*	Young stem nodes, leaf petiole bases, floral peduncles	Elevated oval nectaries; single-layer epidermis; 3–4 layers compact parenchyma; inner parenchyma with phenolic/lipid inclusions; vascularized only by phloem	Secretion released through a central apical pore

## Data Availability

No new data were created or analyzed in this study. Data sharing is not applicable to this article.
